# Aberrant highly prokineticin 2 and its association with inflammatory indexes and functional recovery in acute ischemic stroke patients

**DOI:** 10.3389/fneur.2025.1559688

**Published:** 2025-07-10

**Authors:** Lixiao Sun, Yuxin Fu, Jiecheng Yang, Xiaoli Wang, Dongsheng Cui, Qiang Feng, Xuguang Song, Zhe Li, Zhuangwei Wang, Wenchao Wang, Yiping Wu, Jingmei Wang

**Affiliations:** ^1^Department of Critical Care Medicine, Handan Central Hospital, Handan, China; ^2^Child Care, Merice Cody Public School, Toronto, ON, Canada; ^3^Department of Cardiology, Handan Central Hospital, Handan, China; ^4^Department of Neurology, Handan Central Hospital, Handan, China

**Keywords:** acute ischemic stroke, prokineticin 2, inflammatory status, functional recovery, MRS

## Abstract

**Background:**

Prokineticin 2 is associated with the macrophages-mediated biological process, neuronal death, oxidative stress, and inflammatory processes, while its clinical value in patients with acute ischemic stroke (AIS) has not been explored. This study aimed to evaluate the level of prokineticin 2 and its association with inflammatory indexes and functional recovery in AIS patients.

**Methods:**

Serum samples in 210 AIS patients at admission and in 30 healthy subjects at enrollment were collected. Then, prokineticin 2 levels were determined by enzyme-linked immunosorbent assay.

**Results:**

Prokineticin 2 level was higher in AIS patients than healthy subjects (*p* < 0.001). Prokineticin 2 showed an acceptable ability to distinguish the AIS patients from healthy subjects (area under the curve: 0.812) with the best cut-off value at 4 ng/mL. No matter dividing the prokineticin 2 by continuous variable or quartiles, its value was positively correlated with the high sensitivity C reactive protein (HsCRP), tumor necrosis factor-alpha (TNF-*α*), and interleukin 17A (IL-17A) (all *p* < 0.001). Prokineticin 2 showed a higher trend in AIS patients with Modified Rankin Scale (mRS) score>2 compared with those with mRS score ≤2, but without statistical significance (*p* = 0.095). Besides, there was no association between prokineticin 2 by quartiles and the percentage of mRS > 2 (*p* > 0.05).

**Conclusion:**

Prokineticin 2 aberrantly highly expresses, and it indicates the inflammatory status, but with limited ability to predict the neural functional recovery in AIS patients.

## Introduction

Acute ischemic stroke (AIS), caused by the occlusion of the cerebral artery, is a fatal central nervous system vascular event, which is one of the main reasons for death and disability for human beings worldwide (1–3). During the past decades, with the changes in diet and lifestyle, the incidence of AIS indicates an increased trend, and it is even as high as 13.7 million new cases per year in 2016 (4–6). Therefore, the treatment of AIS has drawn a lot of attention from clinicians recently. Currently, even though some progress has been made in exploring the pathogenesis and the treatment modality for AIS patients [such as thrombolytic, antithrombotic therapy, mechanical thrombectomy (MT), etc.], their prognosis still remains poor due to the short “treatment window” (7–12). Hence, early detection of the pathogenesis of AIS and administrating the corresponding treatment would be helpful in improving the outcomes of AIS patients.

Prokineticin 2, an 8-kD secretory protein, presents a structural similarity with peptide toxins, which has been recently reported to be associated with the macrophages-mediated biological process, neuronal death, oxidative stress, and inflammatory processes (13–16). Recently, the clinical value of prokineticin 2 in the diagnosis of nervous system or inflammation-related disease and evaluation of the disease severity has been preliminary explored (17–19). For instance, one study indicates that prokineticin 2 is highly expressed in Parkinson’s disease patients and serves as a biomarker in indicating gut inflammation (17). Hence, it is reasonable to hypothesize that prokineticin 2 could also be a biomarker to indicate the AIS occurrence and its inflammatory level. However, there is still a lack of evidence.

Hence, this study aimed to explore the level of prokineticin 2 and its association with inflammatory indexes and functional recovery in AIS patients.

## Materials and methods

### Subjects

210 AIS patients between 2020/5 and 2022/9 were included in this study. The inclusive criteria were: (i) Diagnosed as AIS at first time. (ii) Age above 18 years old. (iii) Survived during hospitalization. The exclusive criteria were: (i) Recurrent stroke. (ii) History of systemic inflammatory diseases. (iii) Complicated with infection at the time of blood collection. In addition, another 30 healthy subjects were also included, who were required to age and sex matched to AIS patients. In detail, the age of the healthy control was limited to approximately 65 years, and the sex ratio was limited to the ratio of male to female to 3:2. The protocol was approved by the Ethics Review Board with the approval number of HDSZXYY2019071202, the subjects agreed with the informed consent.

### Serum prokineticin 2 level

Serum samples from AIS patients were collected at the time of admission, regardless of fasting status. Meanwhile, the serum sample from healthy subjects was obtained at enrollment regardless of fasting status. Serum prokineticin 2 level was then detected using a commercial enzyme linked immunosorbent assay kit (Cat no. LS-F22124, LifeSpan Biosciences, USA, range: 0.313–20 ng/mL, sensitivity: 0.188 ng/mL). The intra-assay CV of prokineticin 2 kit was less than 5.77%, and inter-assay CV of prokineticin 2 kit was less than 7.0%.

### Inflammatory marker levels

High sensitivity C reactive protein (HsCRP) level was retrieved from Electronic Medical Record System of hospital. Tumor necrosis factor alpha (TNF-*α*) (Cat no. PT518, Beyotime, China, range: 31.25–1,000 pg./mL, sensitivity: 14.3 pg./mL) and interleukin 17A (IL-17A) (Cat no. PI550, Beyotime, China, range: 31.25–1,000 pg./mL, sensitivity: 11.3 pg./mL) levels were detected using commercial enzyme linked immunosorbent assay kits. The intra- and inter-assy CVs of TNF-*α* and IL17-A kits were less than 10%. Besides, the HsCRP and TNF-α/IL-17A were analyzed from the same sample batches, which were all obtained at admission.

### Data collection

Demographics, medical histories, AIS disease severity and treatment types were collected in AIS patients. Modified Rankin Scale (mRS) was evaluated at 3 months (M3), allowing a visiting window ± 0.5 months. mRS score >2 at M3 was defined as a poor recovery of stroke. In this study, 22 (10.5%) patients lost follow up and did not have data of mRS at M3, therefore not included in the analysis of mRS data.

### Statistical analysis

IBM SPSS Statistics 22 (IBM, USA) was applied for statistical analysis. Comparison of serum prokineticin 2 level between AIS patients and healthy subjects, and between AIS patients with mRS > 2 and those with mRS ≤ 2 at M3, was analyzed by Wilcoxon rank sum test. The comparison of age between AIS patients and healthy controls was carried out by the Student t test, and the comparison of sex between AIS patients and healthy controls was carried out by the chi-square test. Receiver operator characteristic (ROC) curve of serum prokineticin 2 level was drawn to differentiate AIS patients from healthy subjects, and AIS patients with mRS > 2 from those with mRS ≤ 2 at M3. Correlation of serum prokineticin 2 level/quartiles with HsCRP, TNF-*α*, and IL-17A was analyzed by Spearman correlation test, and adjusted by the Bonferroni correction test. Correlation of serum prokineticin 2 quartiles with percentage of mRS > 2 was analyzed by Spearman correlation test. Sensitivity analysis was conducted using extreme scenario simulation methods. In detail, these patients who were lost to follow-up were filled up with an mRS score >2 (poor recovery of stroke). Multivariable logistic regression was carried out to confirm the independent factors correlating with mRS score>2. *p* value below 0.05 was defined as statistical significance.

## Results

### AIS patients’ characteristics

Among the included 210 AIS patients, they had a median [interquartile range (IQR)] age of 65.0 (59.8–73.0) years, with 129 (61.4%) males. Thirty-eight (18.1%) patients had a history of myocardial infarction, and their median (IQR) National Institute of Health stroke scale (NIHSS) score was 9.0 (5.0–13.0). In terms of the treatment modality, there were, respectively, 32 (15.2%) patients receiving recombinant tissue plasminogen activator/alteplase (rtPA), 18 (8.6%) patients receiving tenecteplase (TNK-tPA), 21 (10.0%) patients receiving urokinase (UK) intravenous thrombolysis (IVT), 43 (20.5%) patients receiving rtPA IVT and MT, 27 (12.9%) patients receiving TNK-tPA IVT + MT, 23 (11.0%) patients receiving UK IVT and MT, 46 (21.9%) patients receiving MT. Furthermore, the HsCRP, TNF-*α*, and IL-17A were 3.0 (1.4–4.8) mg/L, 44.3 (36.8–70.2) pg./mL, and 54.6 (39.6–78.5) pg./mL, respectively. Other information is shown in Table 1.

**Table 1:** AIS patients’ characteristics.

Items	AIS patients (*N* = 210)
Age (years), median (IQR)	65.0 (59.8–73.0)
Sex (males), *n* (%)	129 (61.4)
BMI (kg/m^2^), median (IQR)	24.6 (22.4–27.0)
Smoke, *n* (%)	75 (35.7)
History of hypertension, *n* (%)	154 (73.3)
History of hyperlipidemia, *n* (%)	92 (43.8)
History of diabetes, *n* (%)	60 (28.6)
History of myocardial infarction, *n* (%)	38 (18.1)
Period between symptom and admission (hours), median (IQR)	5.0 (4.0–6.0)
NIHSS score, median (IQR)	9.0 (5.0–13.0)
Treatment type, *n* (%)	
rtPA IVT	32 (15.2)
TNK-tPA IVT	18 (8.6)
UK IVT	21 (10.0)
rtPA IVT and MT	43 (20.5)
TNK-tPA IVT + MT	27 (12.9)
UK IVT and MT	23 (11.0)
MT	46 (21.9)
Inflammatory markers, median (IQR)	
HsCRP (mg/L),	3.0 (1.4–4.8)
TNF-α (pg/mL)	44.3 (36.8–70.2)
IL-17A (pg/mL)	54.6 (39.6–78.5)

AIS, acute ischemic stroke; BMI, body mass index; NIHSS, National Institute of Health stroke scale; rtPA, recombinant tissue plasminogen activator/alteplase; TNK-tPA, tenecteplase; UK, urokinase; IVT, intravenous thrombolysis; MT, mechanical thrombectomy; HsCRP, high sensitivity C reactive protein; TNF-α, tumor necrosis factor alpha; IL-17A, interleukin 17A; IQR, interquartile range.

### Comparison of prokineticin 2 between healthy subjects and AIS patients

**Figure 1:**
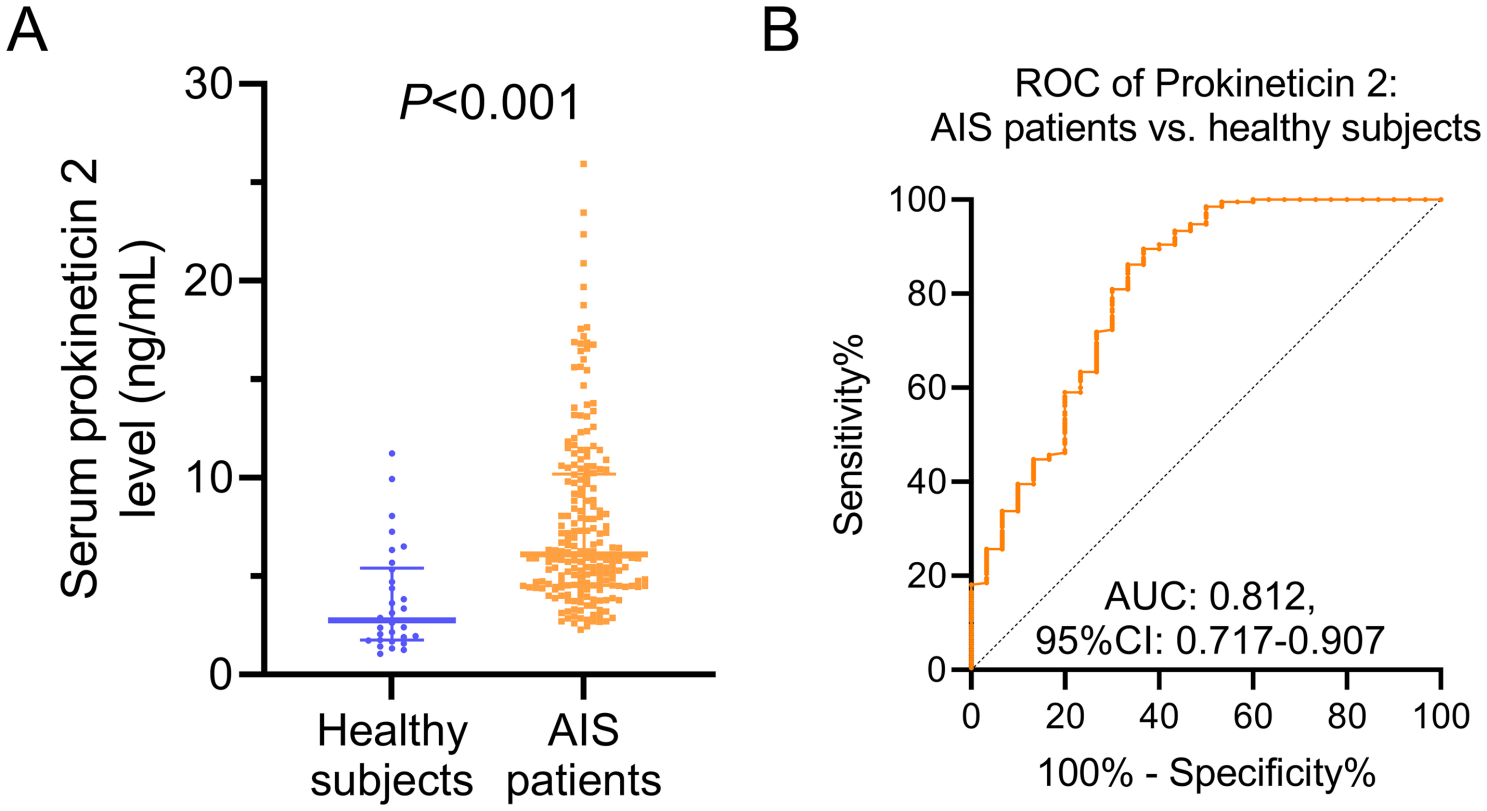
Prokineticin 2 in AIS patients and healthy subjects. Comparison of prokineticin 2 between AIS patients and healthy subjects **(A)**. The ability of prokineticin 2 to distinguish the AIS patients from healthy subjects **(B)**.

In AIS patients, their prokineticin 2 level was higher than that in healthy subjects (*p* < 0.001, Figure 1A). Furthermore, the utility of prokineticin 2 levels in distinguishing the AIS patients from the healthy subjects was acceptable with an area under curve (AUC) [95% confidential interval (CI)] of 0.812 (0.717–0.907) (Figure 1B). Besides, different thresholds of prokineticin 2 levels were applied to explore the best cut-off value for separating the AIS patients from healthy subjects. It was shown that prokineticin 2 level at 4 ng/mL exhibited the highest Yonden index (0.51) with a sensitivity of 0.84 and a specificity of 0.67 (Table 2). There was no difference of the baseline demographics between the AIS patients and healthy subjects (both *p* > 0.05, Supplementary Table 1).

**Table 2:** Thresholds of serum prokineticin 2 level.

Thresholds of prokineticin 2	Sensitivity	Specificity	Likelihood ratio	Youden index
2 ng/mL	1.00	0.33	1.50	0.33
3 ng/mL	0.94	0.53	2.01	0.47
4 ng/mL	0.84	0.67	2.53	0.51
5 ng/mL	0.68	0.73	2.54	0.41
6 ng/mL	0.51	0.80	2.57	0.31
7 ng/mL	0.41	0.87	3.11	0.28
8 ng/mL	0.34	0.90	3.43	0.24

### Correlation of prokineticin 2 with the inflammatory indexes

**Figure 2:**
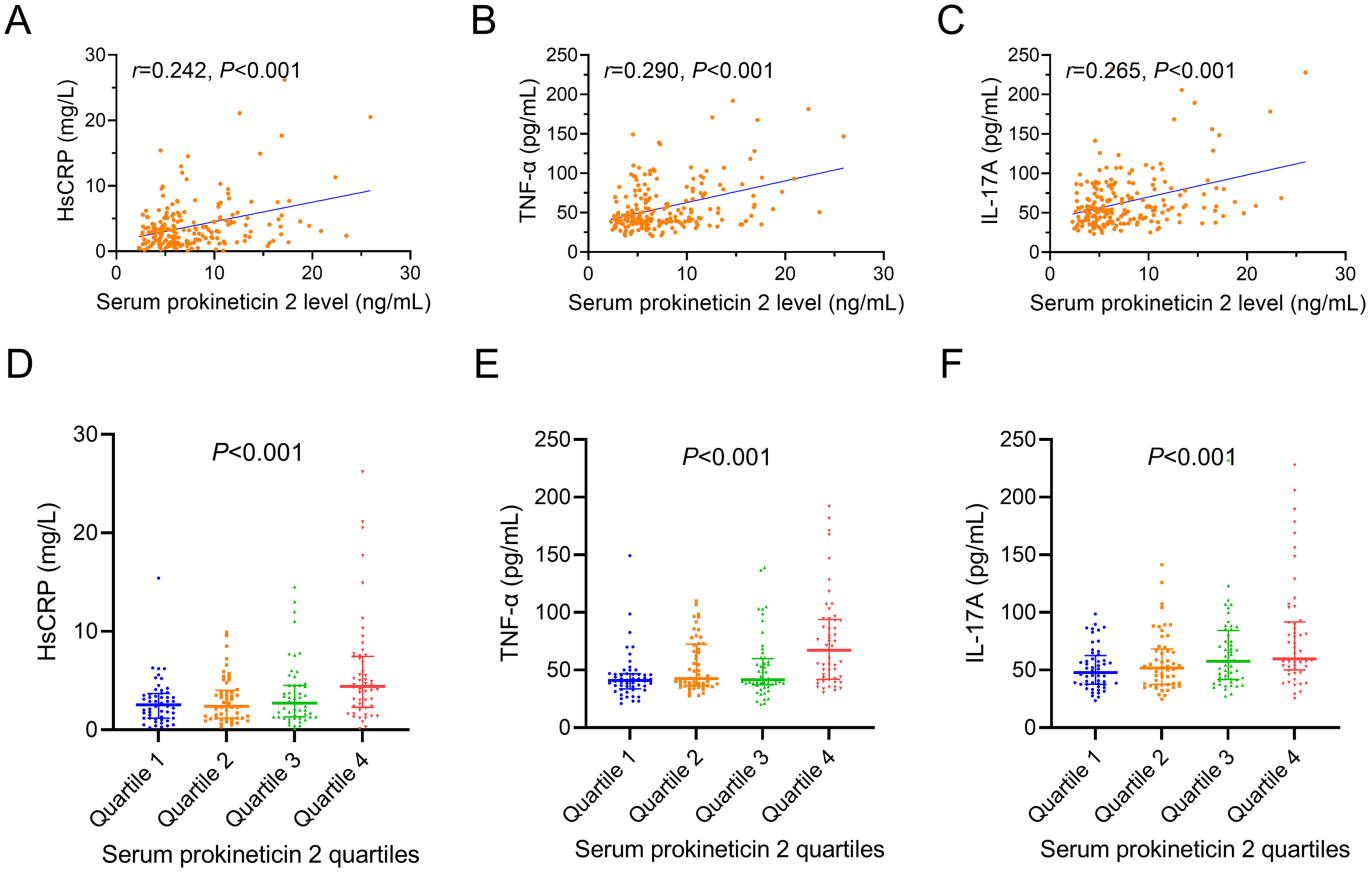
Correlation of prokineticin 2 with the inflammatory indexes. Association of prokineticin 2 (by continous variable) with HsCRP **(A),** TNF-*α* **(B)**, IL-17A **(C)**. Correlation of prokineticin 2 (by quartiles) with HsCRP **(D),** TNF-α **(E)**, IL-17A **(F)**.

The prokineticin 2 was positively correlated with the HsCRP (*r* = 0.242, *p* < 0.001, Figure 2A), TNF-*α* (*r* = 0.290, *p* < 0.001, Figure 2B), and IL-17A (*r* = 0.265, *p* < 0.001, Figure 2C). Subsequently, after dividing the prokineticin 2 by quartiles, it also showed a positive correlation with HsCRP (*p* < 0.001, Figure 2D), TNF-α (*p* < 0.001, Figure 2E), and IL-17A (*p* < 0.001, Figure 2F).

### Association of prokineticin 2 with the functional recovery

**Figure 3:**
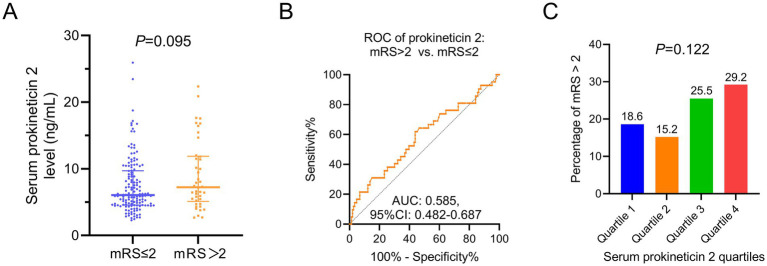
The ability of prokineticin 2 in estimating the neural functional recovery. Comparison of prokineticin 2 between AIS patients with mRS ≤ 2 and >2 **(A)**. The ability of prokineticin 2 to distinguish the AIS patients with mRS ≤ 2 from >2 **(B)**. Correlation of prokineticin 2 (by quartiles) with percentage of AIS patients with mRS > 2 **(C)**.

Prokineticin 2 showed a higher trend in AIS patients with mRS score>2 compared with those with mRS score≤2, but without statistical significance (*p* = 0.095, Figure 3A). The further ROC analysis indicated that the ability of prokineticin 2 in separating the AIS patients with mRS score>2 compared with those with mRS score≤2 was weak [AUC (95% CI): 0.585 (0.482–0.687)] (Figure 3B). Besides, there was no association between prokineticin 2 by quartiles and the percentage of mRS > 2 (Figure 3C). According to the sensitivity analysis, the statistical significance of the correlation between prokineticin 2 and mRS score remained unchanged, indicating the robustness of the main findings (Supplementary Figures 1A–C). Further multivariable logistic regression analysis indicated that prokineticin 2 was not an independent factor associated with the mRS score>2 (Supplementary Table 2).

## Discussion

There were several interesting findings in our study. (1) The prokineticin 2 level was higher in AIS patients compared with the healthy subjects, and it could sperate the AIS patients from the HCs with the best cut-off value at 4 ng/mL; (2) Prokineticin 2 was positively associated with the inflammatory indexes such as the HsCRP, TNF-*α*, and IL-17A; (3) The value of prokineticin 2 in estimating the functional recovery was limited in AIS patients.

The dysregulated expression of prokineticin 2 has been implicated in various pathological contexts, yet its biological roles appear highly disease-specific. For instance, prokineticin 2 is markedly upregulated in neurogenic regions of patients with early-stage Parkinson’s disease, suggesting a role in neuroinflammation or neurodegeneration (17). Conversely, prokineticin 2 levels are reduced in conditions such as Kawasaki disease-a self-limiting vasculitis-and neonatal necrotizing enterocolitis, both of which involve systemic inflammation (18, 20). In our current study, we observed elevated prokineticin 2 levels in patients with AIS, in contrast to these inflammatory disorders. These contrasting patterns suggest that prokineticin 2 may not function as a uniform pro- or anti-inflammatory mediator across diseases. Instead, its role likely depends on the cellular milieu, disease phase, and immune context. For example, in AIS and other neurovascular conditions, prokineticin 2 may contribute to neuroinflammatory cascades and vascular injury. In contrast, in self-limited systemic inflammatory diseases, reduced prokineticin 2 could reflect a feedback mechanism to dampen excessive immune responses. Therefore, rather than attributing a generalized “aggressor” or “defender” function to prokineticin 2, future mechanistic studies should focus on delineating its context-dependent signaling pathways and interactions with disease-specific immune networks in AIS. Besides, even though the ROC curve (AUC = 0.812) demonstrated that prokineticin 2 had acceptable discriminatory power between AIS patients and healthy controls. However, the specificity (67%) at the optimal cutoff (4 ng/mL) was relatively low; this finding suggested that there is still a long way to go to apply the prokineticin 2 in the clinical practice as an early diagnosis biomarker. Furthermore, more biomarkers might need to be explored, and the combination of these biomarkers might further elevate the specificity in discriminating the AIS patients from the healthy controls.

Prokineticin 2 participates in the macrophages-mediated biological process, oxidative stress, and inflammatory processes (13–16, 21, 22), which implies its potential to be an inflammatory marker. However, in the previous studies, prokineticin 2 seems not correlated with the inflammatory cytokine TNF-*α* (23, 24). In the current study, it was found that prokineticin 2 was positively correlated with the inflammatory indexes in AIS patients. These findings could be explained that: (1) Prokineticin 2 activated the inflammation via the calcium-sensing receptor-related pathway (25); (2) Prokineticin 2 could activate the NLRP3 inflammasome pathway in the macrophages, therefore, inducing the inflammatory process (26). Hence, in this study, it was hypothesized that prokineticin 2 also played a proinflammatory role in AIS, thereby positively associated with the proinflammatory indexes.

Another interesting finding in our study should not be neglected: Prokineticin 2 only showed a trend to correlate with the neural functional recovery, but it did not reach statistical significance. These findings seemed contradictory to the previous *in-vivo* study (27). Previously, the prokineticin 2 had been reported to be associated with the neural impairment (27). For instance, the injection of recombinant prokineticin 2 into the cortex of mice would attract subventricular zone cells, which further lead to neural impairment (27). In our opinion, these inconsistent findings might derive from the fact that in real clinical practice, neural functional recovery might be affected by various aspects, such as post-stroke care, diet, physical practice, drug administration, etc. Even though prokineticin 2 might affect neural cell survival in the early stage after stroke, the functional recovery reflected by the mRS score in AIS patients would be mainly affected by other conditions after the acute phase of AIS. Hence, prokineticin 2 was not associated with functional recovery in AIS patients. Beside that, the *p* value of the correlation of prokineticin 2 with mRS > 2 score was 0.095, which almost reach the statistical significance. This phenomen might because sample size and confounding factors. Similar to a recent study reporting that the prokineticin 2 is highly expressed in the neurodegenerative disease patients (such as the Parkinson’s Disease) (17). Another potential explanation for the high expression of prokineticin 2 in AIS patients, while there is a lack of association with functional recovery, might be that prokineticin 2 might play a dual role in neuroinflammation, including its pro-inflammatory effects and neuroprotective effects, simultaneously. For instance, recent studies indicate that prokineticin 2 could prevent the neuronal cell deaths, meanwhile another study reports that prokineticin 2 serves as a proinflammatory peptide (14, 28).

Several limitations in this study were notable: (1) The median age of AIS patients was 65.0 years, which was relatively old. Hence, the study findings might not be suitable for those AIS patients with younger age. (2) Many factors would affect the post-stroke neural functional recovery, which might be the reason that the prokineticin 2 did not associate with the mRS score; hence, further study was needed to include more variables to determine the factors that influent the mRS score in AIS patients. (3) The deep mechanism was not clear about the prokineticin 2 in regulating the pathogenesis of AIS. Hence, further *in vivo* and *in vitro* studies were needed. (4) The subjective bias existed in the evaluation of the mRS score, while the further study would evaluate these indicators such as the NIHSS score and imaging data. (5) The sample size of healthy controls was small, which might affect the reliability of the results. (6) This study was not registered on the public Clinical Trial Registry platform, which was another limitation.

## Conclusion

In conclusion, prokineticin 2 indicates the potential as an inflammatory marker but with limited ability to predict the neural functional recovery in AIS patients. Further study is needed to validate the finding in the current study.

## Data Availability

The original contributions presented in the study are included in the article/Supplementary material, further inquiries can be directed to the corresponding authors.

## Supplementary material

SUPPLEMENTARY FIGURE 1 Sensitivity analysis of the ability of prokineticin 2 in estimating the neural functional recovery by the extreme scenario simulation methods. Comparison of prokineticin 2 between AIS patients with mRS≤2 and >2 **(A)**. The ability of prokineticin 2 to distinguish the AIS patients with mRS≤2 from >2 **(B)**. Correlation of prokineticin 2 (by quartiles) with percentage of AIS patients with mRS>2 **(C)**.
