# Supplementary figures and images for: Aberrant highly prokineticin 2 and its association with inflammatory indexes and functional recovery in acute ischemic stroke patients
Source: Front Neurol. 2025 Jul 10;16:1559688. doi: 10.3389/fneur.2025.1559688 (PMC12286830; doi:10.3389/fneur.2025.1559688)

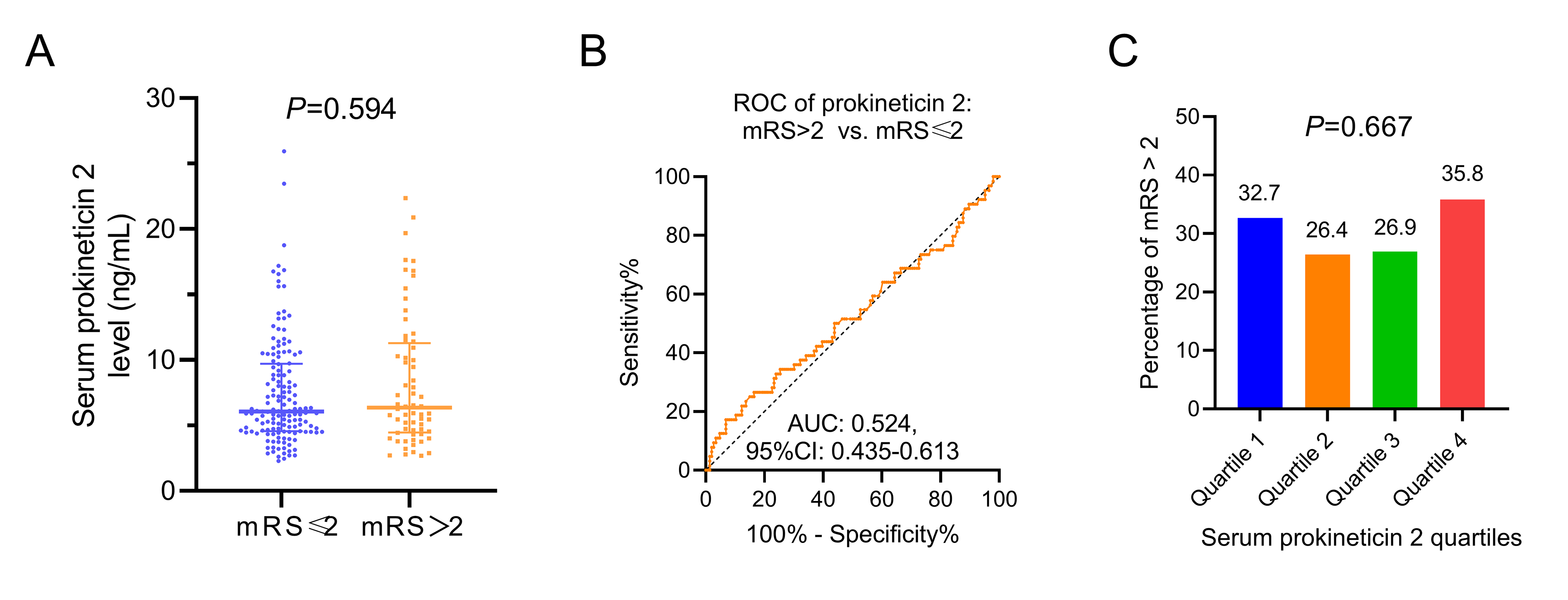

Supplement: SUPPLEMENTARY FIGURE 1 — Sensitivity analysis of the ability of prokineticin 2 in estimating the neural functional recovery by the extreme scenario simulation methods. Comparison of prokineticin 2 between AIS patients with mRS≤2 and >2 (A). The ability of prokineticin 2 to distinguish the AIS patients with mRS≤2 from >2 (B). Correlation of prokineticin 2 (by quartiles) with percentage of AIS patients with mRS>2 (C). [file Image_1.tif]
