# Supplementary material for: Aberrant highly prokineticin 2 and its association with inflammatory indexes and functional recovery in acute ischemic stroke patients
Source: Front Neurol. 2025 Jul 10;16:1559688. doi: 10.3389/fneur.2025.1559688 (PMC12286830; doi:10.3389/fneur.2025.1559688)
Supplement: Supplementary file 2 [file Table_1.docx]

**Supplementary Table 1.** Age and gender between AIS patients and healthy subjects.

| Items | AIS patients (N=210) | Healthy subjects (N=30) | P value |
| --- | --- | --- | --- |
| Age (years), median (IQR) | 65.0 (59.8-73.0) | 65.5 (56.5-72.0) | 0.452 |
| Sex (males), n (%) | 129 (61.4) | 18 (60.0) | 0.881 |

AIS, acute ischemic stroke; IQR, interquartile range.
