# Supplementary material for: Aberrant highly prokineticin 2 and its association with inflammatory indexes and functional recovery in acute ischemic stroke patients
Source: Front Neurol. 2025 Jul 10;16:1559688. doi: 10.3389/fneur.2025.1559688 (PMC12286830; doi:10.3389/fneur.2025.1559688)
Supplement: Supplementary file 3 [file Table_2.docx]

**Supplementary Table 2.** Multivariable logistic regression for mRS score>2.

| Parameters | *P* value | OR | 95%CI of OR | |
| --- | --- | --- | --- | --- |
|  |  |  | Lower | Higher |
| Serum prokineticin 2 | 0.849 | 1.010 | 0.912 | 1.118 |
| Age | 0.639 | 0.988 | 0.939 | 1.039 |
| Sex (males) | 0.454 | 1.417 | 0.569 | 3.530 |
| BMI | 0.877 | 0.991 | 0.878 | 1.117 |
| Smoke | 0.867 | 1.083 | 0.425 | 2.762 |
| History of hypertension | 0.691 | 0.813 | 0.294 | 2.254 |
| History of hyperlipidemia | 0.050 | 2.417 | 1.000 | 5.846 |
| History of diabetes, | 0.275 | 1.648 | 0.672 | 4.038 |
| History of myocardial infarction | 0.046 | 2.801 | 1.019 | 7.700 |
| Period between symptom and admission | 0.179 | 0.785 | 0.552 | 1.117 |
| NIHSS score, median | 0.006 | 1.109 | 1.030 | 1.195 |
| Treatment type |  |  |  |  |
| rtPA IVT | Reference |  |  |  |
| TNK-tPA IVT | 0.046 | 0.076 | 0.006 | 0.954 |
| UK IVT | 0.562 | 0.523 | 0.058 | 4.677 |
| rtPA IVT and MT | 0.357 | 0.414 | 0.063 | 2.703 |
| TNK-tPA IVT+MT | 0.515 | 0.535 | 0.081 | 3.516 |
| UK IVT and MT | 0.304 | 0.373 | 0.057 | 2.446 |
| MT | 0.312 | 0.380 | 0.058 | 2.481 |
| HsCRP | 0.639 | 1.044 | 0.873 | 1.247 |
| TNF-α | 0.348 | 1.011 | 0.988 | 1.036 |
| IL-17A | 0.074 | 0.986 | 0.970 | 1.001 |

mRS, modified Rankin scale; OR, odds ratio; CI, confidence interval; BMI, body mass index; NIHSS, National Institute of Health stroke scale; rtPA, recombinant tissue plasminogen activator/alteplase; TNK-tPA, tenecteplase; UK, urokinase; IVT, intravenous thrombolysis; MT, mechanical thrombectomy; HsCRP, high sensitivity C reactive protein; TNF-α, tumor necrosis factor alpha; IL-17A, interleukin 17A
